# The Book World of Medicine and Science

**Published:** 1896-12-26

**Authors:** 


### The Book World of Medicine and Science.

Lectures on Renal and Urinary Diseases. By Robert Saundby, M.D., F.R.C.P. Price 10s. 6d. net. (Bristol: John Wright and Co. 1896.)

Dr. Saundby has done well to combine in one volume his lectures on Bright's disease and those on diabetes. These were well received at the time of their publication, and the addition of a few chapters on miscellaneous affections of the kidneys has made them into a most useful work on the whole subject. After a short chapter on general anatomy, in which this subject is clearly dealt with, there come several on what we may call symptomatic pathology, the pathology of those various conditions such as albuminuria, dropsy, the production of tube casts, cardio-vascular changes, polyuria, uræmia, and changes in the retina, which together make up the symptomology of Bright's disease. In regard to the vexed question of functional albuminuria, Dr. Saundby is quite clear that albuminuria may be present in healthy persons, and may persist for long periods, without causing any derangement of the general health or of the structure of the kidneys. In regard to the cardio-vascular changes so commonly found in Bright's disease, he thinks that the cardiac hypertrophy is due to stimulation of the heart to increased activity by the presence of non-eliminated poisonous matter, *e.g.*, urea, in the blood, and to capillary resistance; while the capillary resistance may be ascribed to alterations in the density of the blood plasma. In all this one cannot but feel that there is a good deal that is somewhat vague, and that the explanation is not quite satisfying. Still, in saying this of Dr. Saundby, one must admit that much the same might equally fairly be said of the results arrived at by other investigators, for the Newton has not yet arisen to satisfactorily account for all the cardio-vascular phenomena of Bright's disease. The chapter on the clinical examination of the urine gives a fair description of the ordinary methods commonly in vogue. It is a little surprising, however, in view of the great prominence which has recently been given to uric acid as a factor in the production of disease, and of the predominating importance attributed by the author to this agent in the etiology of the special malady in question, viz., Bright's disease, to find that the estimation of the amount of uric acid in the urine is treated so lightly as it is; a little over seven lines being the whole space given to the subject. The various chapters on diabetes are very good, and that on treatment exceptionally full of information. A distinction is definitely drawn between "strict diet" and "modified diet," and it is pointed out that while the former can rarely be persisted in for long its effect in controlling glycosuria in any case is a very valuable measure of the severity of the disease, and an indication how far we may go in adding carbohydrates. Attention is usefully drawn to the fact that some of the gluten bread in the market is nothing more or less than a fraud so far as absence of starch is concerned. The author says, "I have quite given up the use of gluten bread, as the best contains nearly 30 per cent of starch; it is always unpalatable and, relatively to its dietetic value, is absurdly dear." Receipts are given for making almond and cocoa-nut cakes. In regard to drugs the palm is given to opium.

Midwifery. Parts 1 and 2. Catechism Series. Pages 78 and 64. 31 illustrations. Price 1s. each part. (Edinburgh: E. and S. Livingstone.)

These volumes belong to the catechism series for medical students published by Messrs. Livingstone. They are of a handy and convenient size for the pocket. The matter is in the form of question answer, and if used in the way such books should be used, namely, as a means of rapidly revising work previously done, they may be of use to the conscientious student who has carefully gone through a good text-book. The danger, however, of this class of book is that it is simply crammed up by the lazy student immediately before an examination, and, under such circumstances, a thorough knowledge of the subject can not be obtained. The information given is, on the whole, well up to date. There are, however, one or two misprints. On page 35, part 1, uterus is printed for ureters, and on page 55 the presentations are wrongly lettered, both in the text and underneath the diagrams, R.O.A. being printed for L.O.P. Some would also cavil at the definition given of a presentation and the description of a breech presentation as præter-natural labour. The treatment of uterine inertia by the introduction of a catheter between the uterus and the membranes, or by irritating the cervix by the fingers should not be recommended. A useful and fairly full account is given of the artificial feeding of infants. Both parts are well illustrated, and for the purposes of revision they will prove of advantage to students.

